# Methodological problem with comparing increases in different measures of body weight

**DOI:** 10.1186/1756-0500-4-145

**Published:** 2011-05-23

**Authors:** Helen L Walls, Anna Peeters, Haider Mannan, Christopher Stevenson

**Affiliations:** 1 Department of Epidemiology & Preventive Medicine, Monash University, Alfred Hospital, Victoria 3004, Australia

## Abstract

**Background:**

A number of studies have compared proportional increases over time in waist circumference (WC) and body mass index (BMI). However this method is flawed. Here, we explain why comparisons of WC and BMI must take into account the relationship between them. We used data from two cross-sectional US surveys (NHANES 1988-94 and 2005-06), and calculated the percentage change in the average BMI and the average WC between the two surveys, comparing the results with a regression analysis of changes in WC relative to BMI.

**Findings:**

The crude percentage change in BMI (5.8%) was marginally greater than for WC (5.1%). But these percentages cannot be directly compared, as the relationship between the measures is described by a regression equation with an intercept term that does not equal zero. The coefficient of time from the regression equation will determine whether or not WC is on average larger for a given BMI at the second compared with the first time point.

**Conclusion:**

Differences in the percentage change in WC and the percentage change in BMI cannot be usefully directly compared. Comparisons of increases in the two measures must account for the relationship between them as described by the regression equation.

## Introduction

A number of studies have compared changes over time in waist circumference (WC), a measure of abdominal obesity, with body mass index (BMI) and suggested that the nature of excess body weight may be changing to one of greater abdominal obesity [1-8]. Abdominal obesity appears to be more strongly correlated with metabolic and cardiovascular risk than BMI [9,10], and thus whether the nature of the obesity epidemic is changing to one of greater abdominal obesity may have important implications for the burden of obesity-related disease. However some studies exploring this question have based their conclusions on different proportional increases in the two measures of excess body weight [3-5,7,8]. This method is flawed, due to the nature of the relationship between WC and BMI.

## Methods

We analysed data from two nationally representative cross-sectional surveys of the US population, the National Health and Nutrition Examination Studies (NHANES), conducted by the US National Center for Health and Statistics. The surveys were conducted during the time periods 1988 to 1994 (NHANES III), and 2005 to 2006. In each survey participants were selected using a multistage, stratified cluster sampling design to be representative of the civilian, non-institutionalised US population.

We analysed in two ways the change in WC and BMI between these two periods. First, we calculated the percentage change in the average BMI and the average WC between the two time points. Second, we fitted a regression equation with WC as the dependent (outcome) variable and BMI and time as predictor variables--along with a number of potential confounder variables [2].

## Results

In our analysis there was a marginally greater crude percentage change in BMI (5.8%) than WC (5.1%), but using regression analysis we demonstrated that increases in WC were in fact greater than expected based on increases in BMI [2]. Differences in the percentage change in BMI and WC do not indicate that one indicator has moved independently further than the other, due to the nature of the relationship between BMI and WC.

One way to understand this is to consider changes in temperature as measured in degrees Fahrenheit (°F) and degrees Celsius (°C). There is a well-defined structural relationship between Fahrenheit (°F) and degrees Celsius (°C), whereas the example we are exploring of body weight is a data-driven regression relationship. However, the example of the two temperature scales provides a clear and simple example of the underlying issue.

The relationship between these measures of temperature is described by:

An increase in temperature from 25°C to 30°C corresponds to an increase from 77°F to 86°F, and the actual increase in temperature is of course identical, just measured on two different scales. But if the proportional increases in these measures are calculated without consideration of the relationship between them, there appears to be a much greater proportional increase in temperature when measured in degrees Celsius (20.0%) than in degrees Fahrenheit (11.7%). The size of the model intercept term relating the measures (in this case, 32) drives the misconception.

To explore this in a more theoretical manner using the relationship between WC and BMI, let's assume the relationship between WC and BMI does not change over time (and is not affected by confounders - assumed throughout these examples for ease of explanation). Such a relationship is based on this regression equation:(1)

In this equation, *μ *is the model intercept term and *α *is the coefficient of BMI. Thus, rearranging (1) to calculate the percentage change in WC gives us:(2)

In this equation, WC_i_, BMI_i _and t_i _are the values of WC, BMI and time at time i. If the model intercept term (*μ*) is zero, the BMI coefficients (*α*) cancel out and the percentage change in WC is equal to the percentage change in BMI. There is a theoretical common zero for BMI and WC--a person with zero BMI would have zero WC--but in reality this zero is never reached. Over the range of observable values BMI and WC generally have a relationship where the model intercept term (*μ*) is not zero. Thus, whether WC increases more or less than BMI is dependent on the value of *μ*.

Let's explore a real-life scenario using estimates from our data [2]. The estimated mean values for BMI and WC were:

*BMI_1 _*= 27.1 kg/m^2^

*BMI_2 _*= 28.7 kg/m^2^

*WC_1 _*= 93.3 cm

*WC_2 _*= 98.1 cm

The BMI increase from 27.1 kg/m^2 ^to 28.7 kg/m^2 ^corresponds to a 5.8% increase and the WC increase from 93.3 cm to 98.1 cm corresponds to an increase of 5.1%. However we cannot directly compare these percentage increases and conclude that the change in BMI is greater than the increase in WC due to the fact that the relationship between the two measures is described by a regression equation with a model intercept term that does not equal zero. In fact, if we fit the regression model specified in equation (1), then we get the following estimates:

*μ *= 37.2

*α *= 2.2

If this were a structural relationship like the temperature example described above, we could subtract 37.2 from WC. Our adjusted WC would then have a zero coinciding with that of BMI so that the *μ *term in equations (1) and (2) would be zero. Hence we could directly calculate a percent increase in WC--in this case 8.5%-- to compare with the 5.8% increase in BMI. There are two problems with this. Firstly, this is a data-driven relationship not a structural relationship so that the value of the *μ *term will depend on the specific data set we are examining and on any co-variates included in the model. Secondly, if our aim is to compare the increase in WC with that in BMI, then we should have a way of testing whether any observed difference between increases in BMI and WC could have arisen by chance.

A better way to explore whether BMI or WC has moved to a greater extent relative to the other is to consider regression equation (1) with the addition of a time variable:(3)

Equation (3) implies that the coefficient of time (γ) will determine whether or not WC is on average larger for a given BMI at the second time point compared with the first time point.

The parameter estimates from our data were:

*μ *= 21.8

*α *= 2.2

*γ *= 0.85

Note the very different estimate of *μ*--21.8 compared to our earlier estimate of 37.2. Equation (3) implies that for a given BMI, the WC at the second time point will be on average 0.85 cm higher than the WC at the first time point. This clearly shows that for a given increase in BMI, the increase in WC is higher than expected, not lower, as predicted by the comparisons of proportional increases in BMI and WC. Further, we can formally test whether the *γ *term is statistically significantly different to zero--that is whether the higher increase in WC is due to chance. In our case the p value associated with *γ *is p < 0.0001, so the relationship is unlikely to have arisen by chance.

This model is theoretical; the real-life application of this model will require adjustments needed for interactions between variables such as BMI and time, and consideration given to non-linear temporal effects. However, it does illustrate the problems arising in a direct comparison of percentage changes in BMI and WC. Further, while our model is data-driven, this issue is not unique to our analysis. For example, Elobeid et al. [1] found a non-zero intercept when relating BMI to WC using a different modelling analysis.

The relationship could equally be described with BMI as the dependent variable and WC as the independent variable. In this case equation (3) would be reformulated as:(4)

Note that one feature of regression analysis is that this will generally not be the same equation as equation (3). The parameter estimates from our data were in this case:

*μ *= -3.66

*α *= 0.37

*γ *= -0.02

Equation (4) implies that for a given WC, the BMI at the second time point will be on average 0.02 kg/m^2 ^smaller than the BMI at the first time point.

## Conclusion

The relationship between the percentage change in WC and the percentage change in BMI is not simple and differences in their percentage increases cannot be directly compared. Comparisons of increases in the two measures must account for the relationship between them as described by the regression equation.

## Competing interests

The authors declare that they have no competing interests.

## Authors' contributions

HLW wrote the article. All authors contributed to formulation of concepts. CS and HM contributed to revisions of the text and CS supervised the analysis. All authors read and approved the final manuscript.
